# MiR-4524b-5p/WTX/β-catenin axis functions as a regulator of metastasis in cervical cancer

**DOI:** 10.1371/journal.pone.0214822

**Published:** 2019-04-02

**Authors:** Tong Li, Wenjuan Zhou, Yimin Li, Yaqi Gan, Yulong Peng, Qing Xiao, Chunli Ouyang, Anqi Wu, Sai Zhang, Jiaqi Liu, Lili Fan, Duo Han, Yu Wei, Guang Shu, Gang Yin

**Affiliations:** 1 Department of Pathology, Xiangya Hospital, School of Basic Medical Sciences, Central South University, Changsha, China; 2 Xiangya School of Nursing, Central South University, Changsha, China; 3 Department of General Surgery, The Third Xiangya Hospital of Central South University, Changsha, China; 4 School of Life Science, Inner Mongolia University, Hohhot, Inner Mongolia Autonomous Region, China; Universitat des Saarlandes, GERMANY

## Abstract

Cervical cancer is the second most deadly gynecological tumor worldwide. MicroRNAs (miRNAs) play very important roles in tumor oncogenesis and progression. The mechanism of post-transcription regulation of WTX gene is still unknown. A series of differential miRNAs were discovered by microarray analysis comparing three pairs of primary cervical cancer specimens and their relapsed tumors from three patients. Quantitative reverse transcriptase PCR (qRT-PCR), Western Blot (WB) and Immunohistochemistry (IHC) was used to detect the expression of miR-4524b-5p and WTX in cervical cell lines and tissues. The biological function of miR-4524b-5p and WTX was investigated through knockdown and overexpression with inhibitor/siRNA and mimic/plasmid in vitro and in vivo. In this study, we found that miR-4524b-5p is highly expressed in relapsed cervical cancer specimens. Combined in vitro and in vivo experiments, showed that miR-4524b-5p could regulate the migration and invasion ability of cervical cancer. Furthermore, we also found that miR-4524b-5p could regulate the migration and invasion of cervical cancer by targeting WTX and that WTX could regulate the expression of β-catenin. Taken together, our data identified a miR-4524b-5p/WTX/β-catenin regulatory axis for cervical cancer, and miR-4524b-5p may be a potential target for cervical cancer therapy.

## Introduction

Cervical cancer is the second most common gynecological cancer and a leading cause of cancer death in women[1]. With improved cervical cancer screening, an increasing number of cervical cancer patients have been diagnosed at an early stage, and the outcomes were improved due to combined cancer therapies[2, 3]. However, cancer recurrence occurs in some cervical cancer patients who receive early diagnosis and radical treatments, and the survival rate declines significantly after cancer recurrence in those patients[4, 5]. Cancer stage, histopathological types or cancer treatments cannot predict whether a patient will be free of disease or will present with recurrent disease; therefore, there is a need for new biomarkers that can help to identify patients at risk of recurrence and consequently assist in diagnosis and treatment decision making[6–8].

MicroRNAs (miRNAs) are small (17–25 nucleotides in size) noncoding RNA molecules that regulate gene expression through several mechanisms[9, 10]. Studies have reported that altered expression of miRNA plays a major role in tumor oncogenesis and progression[11]. Numerous miRNAs have been correlated with cellular proliferation, apoptosis, differentiation, migration, and cancer prognosis[12–15]. Many investigations have shown that miRNA is abnormally expressed in different types of tumors[16], including ovarian cancer[17], non-small-cell lung cancer[18], pancreatic cancer[19]. As a result, the use of miRNA as a tumor molecular biomarker represents an alternative and specific approach for the diagnosis and selection of therapeutic approaches.

Some miRNAs have been found to regulate oncogenes and promote cervical cancer development. For example, the expression of miR-29 is influenced by the cellular microenvironment, which is rebuilt by HPV infection and finally induces oncogenesis[20]. MiR-196a targets FOXO1 and p27 to enhance the proliferation of cervical cancer cells[21]. MiR-23b is in the p53/miR-23b/uPA pathway, which is promoted by HPV16 E6 protein and enhances the migration of cervical cancer cell [22]. As a consequence, miRNAs have unique advantages as targeted molecules in cervical cancer diagnosis and treatment. In this study, we found that miR-4524b-5p is highly expressed in relapsed cervical cancer patients.

WTX, also called Amer1 (APC membrane recruitment protein 1) is the first tumor suppressor gene located on the X chromosomal and was described by Rivera in 2007[23]. Michael B. Major revealed that WTX forms a complex with β-catenin, AXIN1, β-TrCP2, and APC. These authors also demonstrated that WTX promotes β-catenin ubiquitination and degradation in cultured cells, Xenopus, and zebrafish, which antagonize WNT/β-catenin signaling [24]. The overexpression of WTX in human embryonic kidney (HEK) 293 cells and in U2OS human osteosarcoma cells significantly suppressed colony formation[23]. LIU Xia analyzed the promoter methylation levels of WTX and found no significant differences in methylation levels between normal gastric tissues, gastric cancer tissues and 3 gastric cancer cell lines[25]. The mechanisms related to the regulation of WTX expression in cervical cancer are unclear.

We chose three pairs of tumor tissues from three patients who were diagnosed with early-stage cervical cancer and recurrence in 2~3 years. Comparing the primary and relapsed tumors specimens from the same patients, we found that the expression of miR-4524b-5p was highly expressed in relapsed tumor patients. Our results showed that miR-4524b-5p might function as an oncogene by regulating the expression of WTX, a tumor suppressor gene. Using in vitro models, we demonstrated that WTX is a target gene of miR-4524b-5p and that miR-4524b-5p inhibited the expression of WTX by targeting the 3′-UTR of WTX.

## Materials and methods

### Cell culture

HEK-293T and H8 cells were grown in Dulbecco’s modified Eagle’s medium (DMEM) (Gibco, Carlsbad, USA) with 10% fetal bovine serum (FBS) (Gibco, Carlsbad, CA). The cervical cell lines SiHa, HeLa and me-180 were cultured routinely in RPMI-1640 medium (Gibco, Carlsbad, USA) supplemented with 10% FBS, and incubated at 37 ºC with 5% CO_2_.

### Cell transfection

The miRNA mimic, miRNA inhibitor and siRNA were designed and synthesized by RiboBio (Guangzhou, China). The pCDH plasmids encoded the full-length cDNA sequence of WTX. For cell transfection, the miR-4524b-5p mimic, inhibitor, siRNA or control was transfected into cells with Lipofectamine 2000 Reagent (Life Technologies) according to the manufacturer’s protocol.

### RNA extraction and mRNA/miRNA detection

Total RNA was extracted from prepared cells using TRizol reagent (Invitrogen, CA USA). The cDNA of mRNA and miRNA were synthesized with the GoScript Reverse Transcription System (Promega, Madison, USA) or the All-in-One miRNA First Strand cDNA Synthesis Kit (GeneCopoeia, Rockville, USA) according to the protocol. Real-time PCR was performed using the GoTaq qPCR Master Mix (Promega, Madison, USA) with the following primers: WTX forward, 5’-CAGCTCAGGGAGGTTTTGAG-3’ and reverse, 5’-CCAGACATGCAAGAAGCAAA-3'; GAPDH forward, 5’-CAAGGTCATCCATGACAACTTTG-3’ and reverse, 5’-GTCCACCACCCTGTTGCTGTAG-3'. For miRNA detection, we used the All-in-One miRNA qRT-PCR Detection Kit (GeneCopoeia, Rockville, USA). GAPDH or U6 were used as endogenous controls, and the ddCt method was used to analyze relative mRNA or miRNA expression, respectively.

### Western blot analysis

The cell lysates were extracted using RIPA buffer (Beyotime, Haimen, China) and 1% PMSF (Wuhan, China). The extracted protein (30 μg or 50 μg) was separated by sodium dodecyl sulphate polyacrylamide gel (10% polyacrylamide gels, SDS-PAGE), then blotted to PVDF membranes by electroblotting. The membrane was immunoblotted with a primary antibody specific to WTX (Sigma) and β-catenin (Cell Signaling). The signals were developed by using Sage Brightness West Pico plus ECL Solution (Pico Plus). GAPDH (Utibody) was used as an internal control protein.

### Wound-healing assays

A total of 1×10^5^ cells were seeded onto each well of a 6-well plate with 90% confluence in RPMI-1640 containing 10% FBS. When the well was almost full of cells, the cell monolayer was scraped straight away by using 200 μL tips and washed with phosphate buffered saline (PBS) to remove cell debris. The scraped monolayer was incubated in serum-free medium for 48 h, and wound healing areas were measured using a phase contrast microscope (Olympus Corp, Tokyo, Japan). ImageJ Plus was used to quantify the wound-healing assays. Each experiment was repeated three times.

### Cell migration assay

A Transwell assay was performed using a Transwell chamber of 8 μm pore size culture inserts (Corning 3422, NY, USA). A total of 1×10^6^ cells/mL in 200 μL serum-free RPMI-1640 medium were plated in the upper chamber of the insert. The lower chambers of the inserts were incubated in the medium with 15% FBS. Following 12–24 h, cells that had migrated to the bottom of the inserts were immobilized with 4% paraformaldehyde then stained with 0.1% crystal violet for 20 min. Cells were counted using a phase contrast microscope (Olympus Corp, Tokyo, Japan).

### Cell invasion assay

The invasion ability of the cells was determined by Transwell assays. Cell suspensions at 5 × 10^5^ cells/mL were seeded onto the upper chamber coated with Matrigel (BD Biosciences, USA). Culture medium containing 15% FBS was added to the bottom chamber. After incubation for 24 h, invaded cells were fixed in 4% paraformaldehyde, stained with crystal violet and counted. The numbers of invading cells were assessed using a phase contrast microscope (Olympus Corp, Tokyo, Japan).

### Target predictions

We used two miRNA target algorithms: TargetScan (release 7.0, http://www.Targetscan.org/) and miRDB (http://www.mrd.org/miRDB/) for the bioinformatics analysis.

### Plasmid construction

The full length 3’UTR fragment of the WTX gene containing the putative miR-4524b-5p binding site was cloned into the pYr-MirTarget vector by Yingrun (Changsha, China). Mutant WTX 3’UTR (forward primer: 5’-CAAGTGCATCTGCCCAGGGAGGCTGCTGCCATTAC-3’; reverse primer: 5’-AAAAACTGGAGTGCAGTAATGGCAGCAGCCTC-3') was obtained using the Mut Express-II Fast Mutagenesis Kit (Vazyme Biotech Co, Ltd.).

### Dual-luciferase activity assay

HEK-293T cells were seeded in 24-well plates and cultured for 24 h before transfection. The pYr-MirTarget 3′UTR-wild-type (WT) plasmid (0.1 μg) or pYr-MirTarget 3′UTR-mutant (Mut) plasmid together with the 50 nM miR-4524b-5p mimic or miR-Ctrl, were cotransfected into cells with Lipofectamine 2000 (Life Technologies). After 48 h of incubation, cells were harvested. The Firefly and Renilla signals were determined by the Dual-Luciferase reporter assay system (Promega, Madison, USA).

### Patients and samples

Cervical samples, formalin-fixed, paraffin-embedded (FFPE) tissues, were obtained from Hunan Cancer Hospital between 2005 and 2012, of whom we had complete clinical and follow-up data. We obtained written informed consent from all of the patients. This project was approved by the ethics committee of Xiangya Hospital (Central South University, Changsha, China). All specimens were handled according to the ethics committee guidelines.

### Immunohistochemistry (IHC)

The sections from paraffin-embedded blocks were deparaffinized by xylene and dehydrated through a graded alcohol series. Tissues were washed 3 times with PBS for 5 min, and incubated with 3% hydrogen peroxide for 20 minutes. The samples were washed 3 times with PBS for 5 minutes. Antigen retrieval was performed by pressure cooking for 3 minutes in citrate-buffered solution (pH = 6.0). Then, the tissues sections were incubated with 10% goat serum at room temperature for 30 minutes. The sections were incubated with the following primary antibodies: anti-WTX (1:100, Sigma) at 4 ºC overnight. The tissues were incubated at room temperature for 45 minutes and washed with PBS followed by incubation with a secondary goat anti-rabbit antibody conjugated with horseradish peroxidase for 30 min at room temperature. Finally, 3- diaminobenzidine tetrahydrochloride (DAB) was added to the sections, incubated for 2–3 min, and then counterstained in hematoxylin. Finally each sample was scored as negative (0), low (1), medium (2) or high (3) according to staining intensities.

### In vivo tumor xenograft studies and metastasis assays

All animal studies were performed under the protocol approved by the Animal Research Committee of Central South University in accordance with the “Guide for the Care and Use of Laboratory Animals” and the “Principles for the Utilization and Care of Vertebrate Animals”. Ten 4-week-old male BALB/c nude mice were purchased, and maintained under pathogen-free conditions. In our experiments, 3 × 10^6^ cells were subcutaneously injected into the double flanks of one mouse, leading to the formation of two tumors per animal. Tumor diameters were measured every 2 days. Tumor growth was monitored by the tumor volume, which was calculated as follows: volume (mm^3^) = width^2^ (mm^2^) × length (mm) × 1/2. For in vivo metastasis assays, 6 × 10^6^ cells were injected into the intraperitoneal of nude mice. After 8 weeks, the mice were killed, and intraperitoneal metastatic colonization was monitored.

### Statistical analysis

All experiments were repeated at least three times. All statistical analyses were performed using GraphPad Prism software. The statistical significance was determined by Student’s *t*-test. The data are presented as the means ± SEM. Differences were considered statistically significant at *P* < 0.05.

## Results

### High expression of miR-4524b-5p in relapsed human cervical cancer tissues

**Fig 1:**
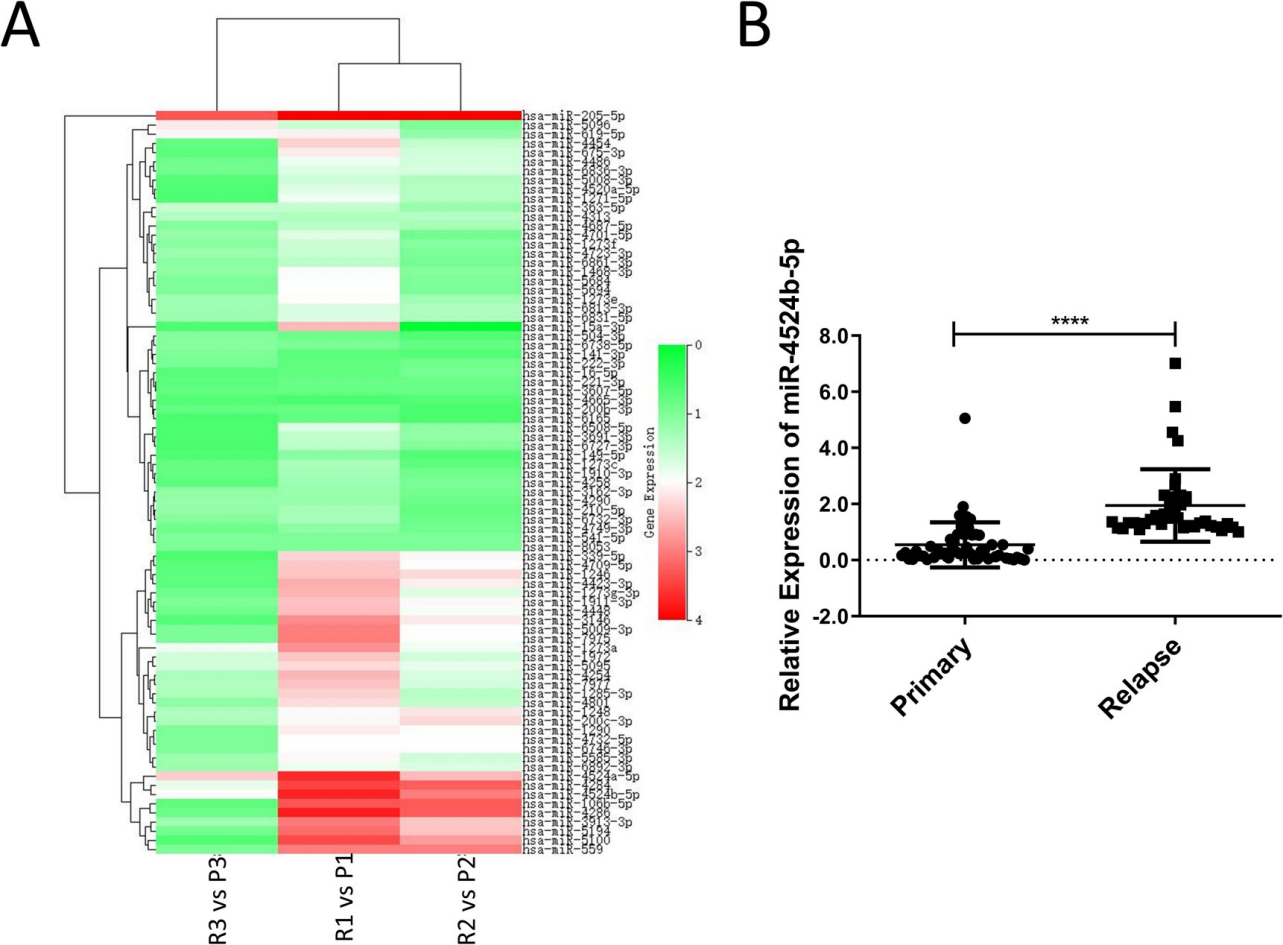
MiR-4524b-5p expression is higher in relapsed cervical cancer specimens than in primary specimens. (A) Heatmap analysis the expression of 81 miRNAs in relapsed and primary cervical cancer tissues; hsa-miR-4524b-5p expression is higher in relapsed cervical tissues (R: relapsed; P: primary). (B) Compared with primary cervical tissues (n = 50), miR-4524b-5p is significantly increased in relapsed cervical tissues (n = 39).

To identify miRNAs associated with relapse and the metastasis of cervical cancer patients, we performed microarrays to evaluate the differential expression of miRNAs in three pairs of tumor samples obtained from three patients who were diagnosed with early-stage cervical cancer and recurrence in 2~3 years (Fig 1A). Eighty-one miRNAs were significantly increased and that hsa-miR-4524b-5p was among the top differentially expressed miRNAs; therefore, we focused on its characterization. Using qRT-PCR, we found that miR-4524b-5p was significantly increased in the relapsed cervical cancer specimens (n = 39) relative to the primary specimens (n = 50) (Fig 1B).

### MiR-4525b-5p promoted the migration and invasion but did not affect the proliferation of SiHa cervical cancer cells in vitro

**Fig 2:**
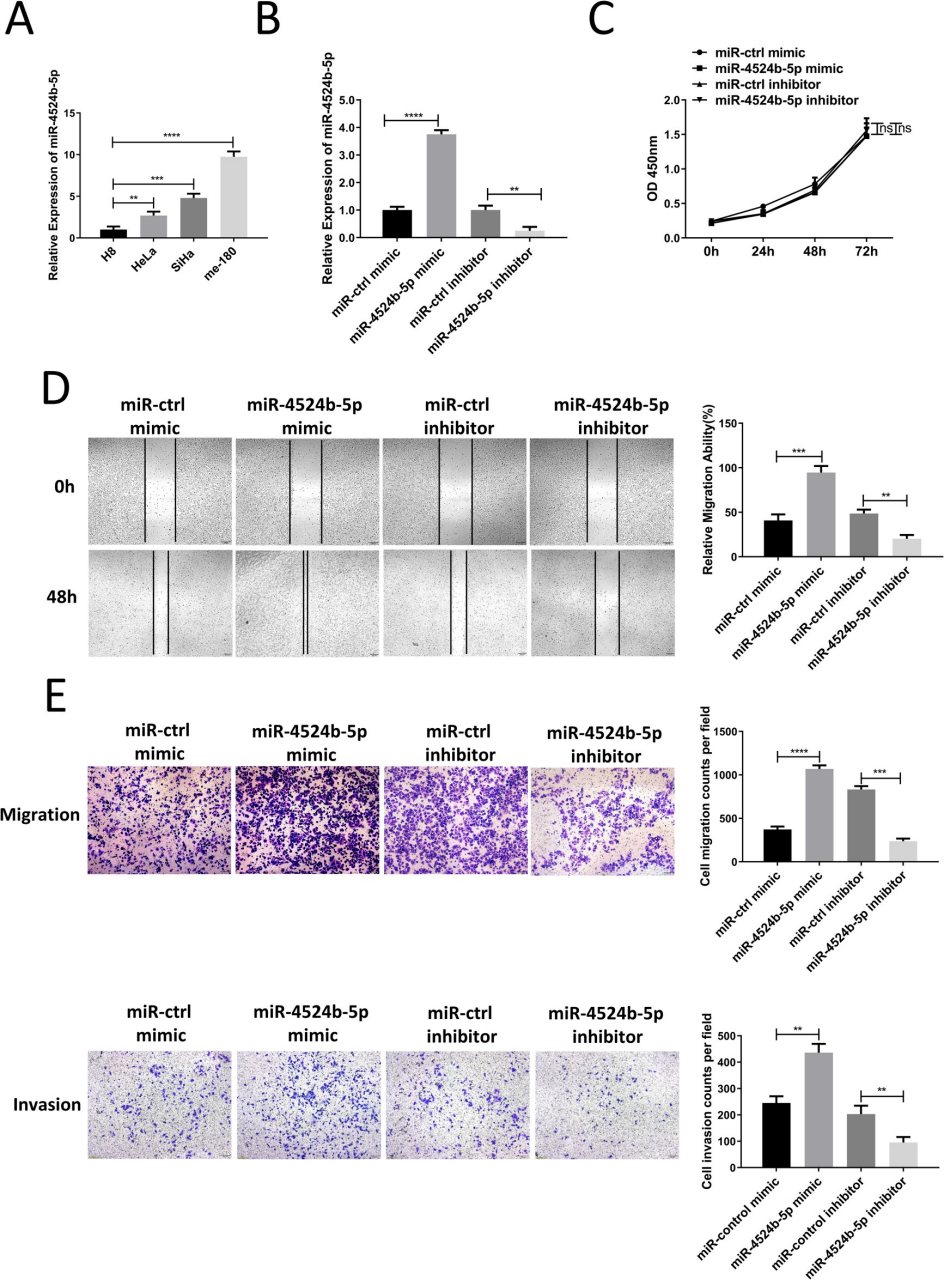
MiR-4524b-5p promoted SiHa cell migration and invasion in vitro, but did not affect its proliferation. (A) Relative expression of miR-4524b-5p in cervical cancer cell lines and normal cell lines. (B) Relative expression of miR-4524b-5p in SiHa transfected with miR-4524b-5p mimic or inhibitor. (C) Cell proliferation was examined using a CCK-8 assay. The proliferation of SiHa cells transfected with miR-4524b-5p mimic/miR-4524b-5p inhibitor compared with that of cells transfected with miR-control mimic/miR-control inhibitor. (D) Wound-healing assay measurement of the migration ability of SiHa cells transfected with miR-4524b-5p mimic or inhibitor for 48 h. (E) Transwell assay measurement of the migration and invasion ability of SiHa cells transfected with miR-4524b-5p mimic or inhibitor for 48 h. All values are presented as the means ± SEM, **P < 0.01, ***P < 0.001, ****P < 0.0001.

To determine the specific effect of miR-4524b-5p on the development and progression of cervical cancer. We detected the expression of miR-4524b-5p in cervical cancer cell lines and normal cell line, and we showed that the expression of miR-4524b-5p was relatively medium in SiHa cells. Therefore this cell lines would be used in the following experiment (Fig 2A). We overexpressed miR-4524b-5p by transfecting SiHa cells with a miR-4524b-5p mimic, and knock down miR-4524b-5p by transfecting SiHa cells with a miR-4524b-5p inhibitor. The alteration of miR-4524b-5p expression was validated by qRT-PCR at 48 h after transfection (Fig 2B). To detect the function of miR-4524b-5p, we analyzed the potential effect of miR-4524b-5p on proliferation. However, there were no significant changes in proliferation ability upon miR-4524b-5p overexpression or inhibition in SiHa cells (Fig 2C). Then miR-4524b-5p overexpressing SiHa cells were subjected to Transwell assays and wound-healing assays, which showed a significant promotion of migration and invasion upon miR-4524b-5p overexpression. Consistent with the overexpression, miR-4524b-5p inhibition resulted in an effective decrease in the migration and invasion of SiHa cells (Fig 2D and 2E). These data showed that miR-4524b-5p promoted the migration and invasion ability of SiHa cells in vitro.

### MiR-4525b-5p promoted the metastasis of cervical cancer in vivo

**Fig 3:**
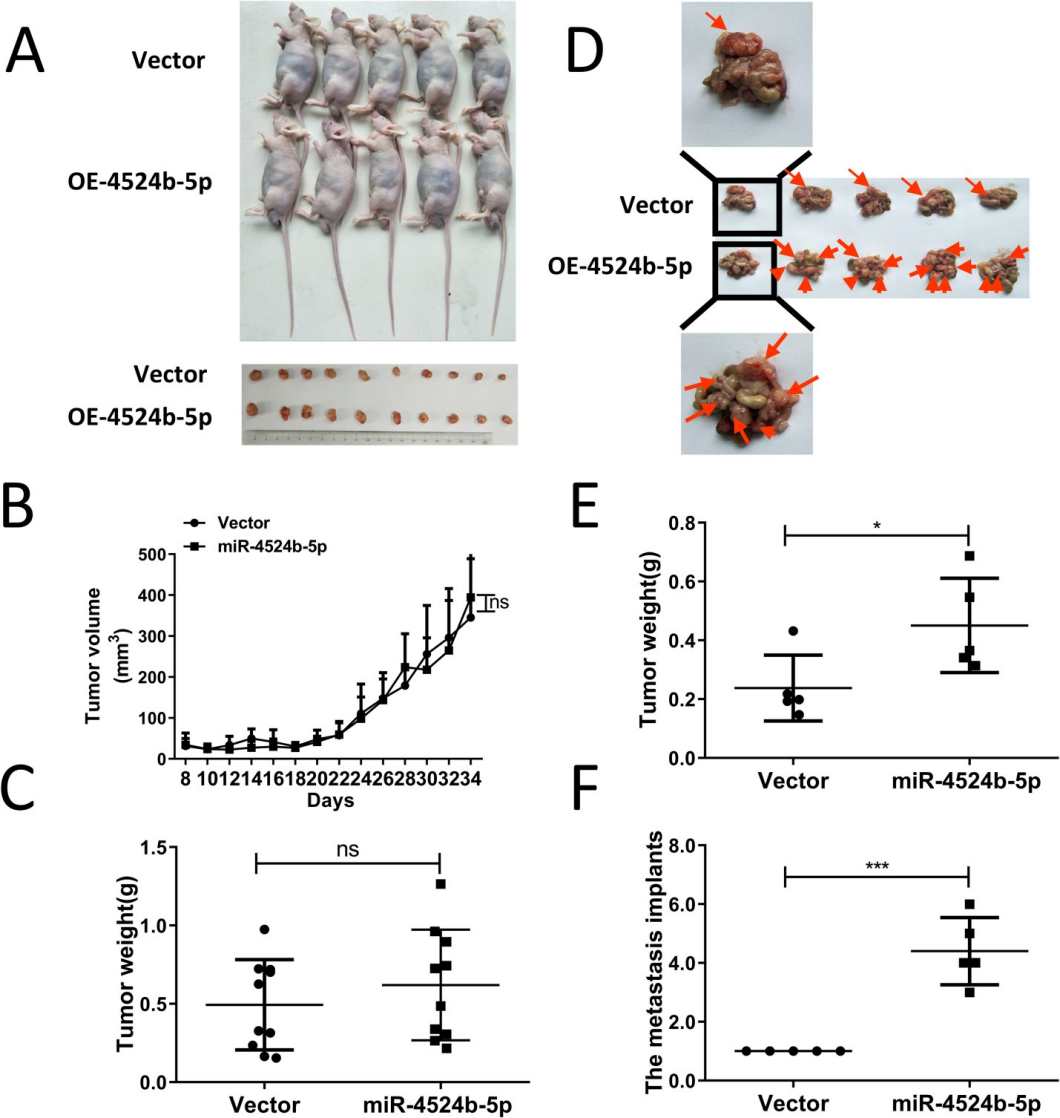
MiR-4524b-5p enhanced metastasis ability of human cervical cancer cells in vivo. (A) Examination of tumorigenesis in animals subcutaneously injected with SiHa cells stably expressing the negative control or miR-4524b-5p. (B) The tumor growth curve of miR-4524b-5p-expressing cells was compared with that of negative control cells. (C) The tumor weight of miR-4524b-5p-expressing cells was compared with that of negative control cells. (D) Mouse abdominal metastatic tumor model (5 mice in each group). Representative photos of abdominal metastatic nodules are indicated by red arrows. (E) The abdomen tumor weight of miR-4524b-5p-expressing cells was compared with that of negative control cells. (F) The metastasis implants of miR-4524b-5p-expressing cells were compared with those of negative control cells. All data are presented as the means ± SEM, *P < 0.05, ***P < 0.001.

We examined the facilitation effects of miR-4524b-5p on the migration of cervical cancer cells in vitro, and then detected the role of miR-4524b-5p in cervical cancer tumor metastasis in vivo. For the comparison of tumor growth, SiHa cells with stable expression of miR-4524b-5p and SiHa-vector cells were inoculated subcutaneously into 5 nude mice on double sides of flanks with 3 × 10^6^ cells per mouse. The animals were closely monitored for tumor growth for 4 weeks, and tumor sizes were measured every two days. The results showed that the tumors from miR-4524b-5p-overexpressing SiHa cells had no significant difference in tumor volume and weight compared with the control cells (Fig 3A–3C). Furthermore, SiHa-miR-4524b-5p and SiHa-vector cells were injected into the abdomen to detect metastasis ability in vivo. At 4 weeks after injection, the peritoneal cavities of the mice were excised to observe more metastasis implants on the surface from miR-4524b-5p overexpressing cells compared to those of mice inoculated with SiHa-vector cells. The tumor scraped from the miR-4524b-5p overexpressing cell intestine was heavier than that from the SiHa-vector cells (Fig 3D–3F). Taken together, these results indicated that miR-4524b-5p overexpression can promote tumor metastasis in vivo.

### MiR-4524b-5p inhibited WTX expression levels

**Fig 4:**
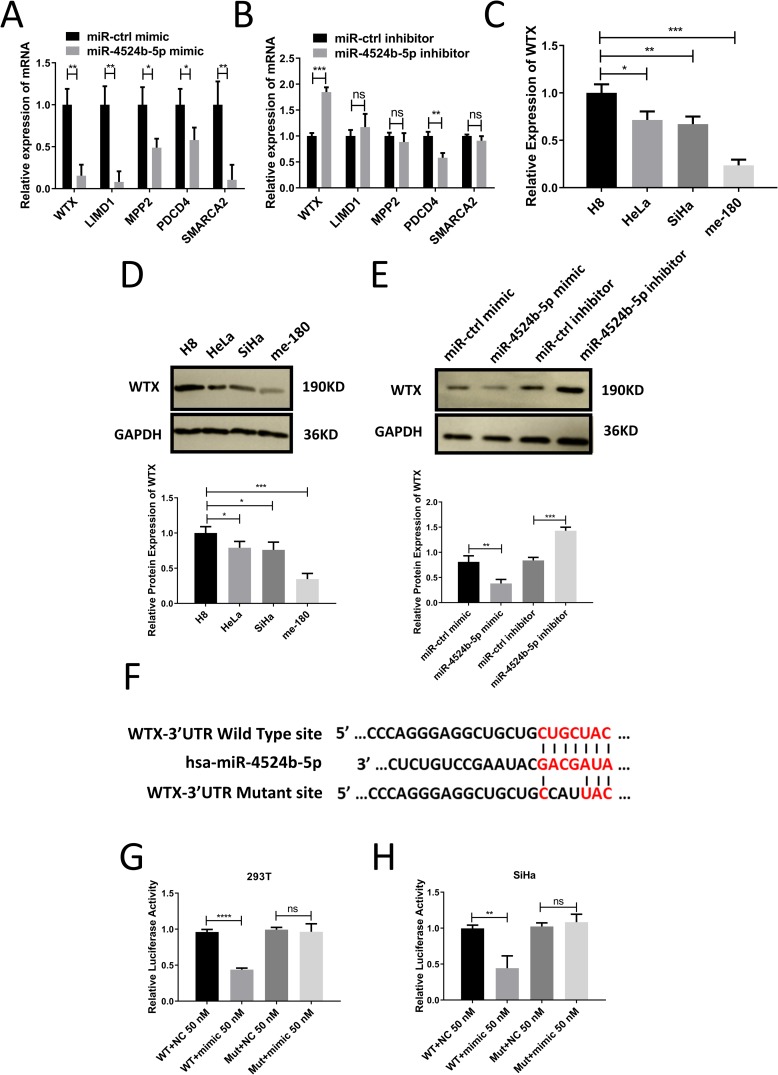
MiR-4524b-5p downregulated WTX mRNA and protein levels by directly targeting the 3’UTR of WTX. (A) Relative expression of five potential gene mRNAs in SiHa transfected with miR-4524b-5p mimic or miR-control mimic. (B) Relative expression of five potential gene mRNAs in SiHa transfected with miR-4524b-5p inhibitor or miR-control inhibitor. (C) Relative mRNA expression of WTX in cervical cancer cell lines and normal cell lines. (D) Relative protein expression of WTX in cervical cancer cell lines and normal cell lines. (E) Western blot analysis of the effects of miR-4524b-5p overexpression and inhibition on WTX protein levels in SiHa cells. (F) The putative binding site in the WTX mRNA 3’UTR for miR-4524b-5p was predicted by Targetscan. (G-H) Relative luciferase activity in HEK-293T and SiHa cells cotransfected with the luciferase-3’UTR construct (wild or mutant type) with the miR-4524b-5p mimic, and the luciferase activities was determined by luciferase reporter assay. All values are presented as the means ± SEM, *P < 0.05, **P < 0.01, ***P < 0.001, ****P < 0.0001.

To understand the potential mechanism of miR-4524b-5p affecting the biological functions of SiHa cells. We used online bioinformatics tools to predict possible target genes of miR-4524b-5p using two online prediction algorithms (TargetScan and miRDB). Then, 126 candidate genes were predicted to be potential targets of miR-4524b-5p by both algorithms. We selected five genes (WTX, LIMD1, MPP2, PDCD4 and SMARCA2) with high scores and evaluated the expression of the five gene mRNAs in SiHa cells transfected with the miR-4524b-5p mimic or the miR-4524b-5p inhibitor. WTX mRNA expression was reduced after miR-4524b-5p overexpression, and WTX mRNA was increased after miR-4524b-5p inhibition (Fig 4A and 4B). Furthermore, we checked the expression of WTX mRNA and protein in cervical cancer cell lines and normal cell line, and determined that the expression of WTX mRNA and protein expression was relatively medium in SiHa cells (Fig 4C and 4D). Western blot assays revealed that the overexpression/inhibition of miR-4524b-5p significantly decreased/increased the expression of WTX protein levels (Fig 4E).

To confirm the predicted consensus sequences for miR-4524b-5p in the WTX 3’-UTR, and determine whether these miR-4524b-5p-binding sequences directly contributed to the negative regulation of WTX expression, we tested the effects of miR-4524b-5p on the activity of a reporter gene using vectors that either contained wild-type or mutant miR-4524b-5p-targeting sequences (Fig 4F). As shown in Fig 4G and 4H, the cotransfection of HEK-293T and SiHa cells with miR-4524b-5p mimic resulted in a significant reduction in the activity of the reporter gene vector containing wild-type 3’-UTR targeting sequences; in contrast, there was only a slight decrease in the activity of the reporter gene vector containing mutant 3’-UTR sequences. These results demonstrated that the miR-4524b-5p-binding sequence in the WTX-3’-UTR was the region required for the miR-4524b-5p-mediated the inhibition of WTX expression.

### Knock down WTX promoted the migration and invasion of cervical cancer cells

**Fig 5:**
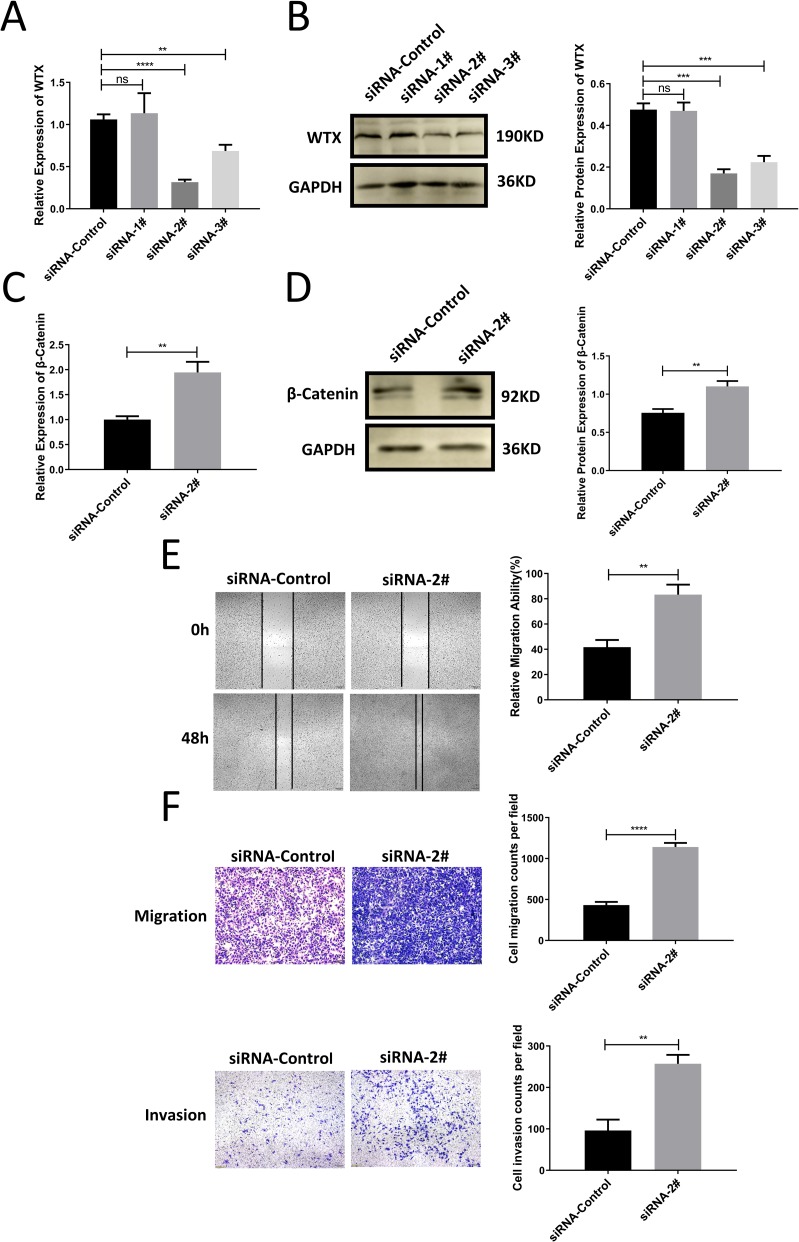
Knock down WTX promoted cervical cancer cell migration and invasion. (A-B) WTX expression in SiHa cells transfected with siWTX-1#, 2#, 3# and siRNA-control. Changes in mRNA abundance were determined by qRT-PCR. Changes in protein abundance were determined by western blotting. (C-D) β-catenin expression in SiHa cells transfected with siWTX-2# and siRNA-control. Changes in mRNA abundance were determined by qRT-PCR. Changes in protein abundance were determined by western blotting. (E) Cell migration was analyzed using a wound-healing assay. The migration of SiHa transfected with siWTX-2# was increased, compared with that of cells transfected with siRNA-control. (F) Cell migration and invasion were analyzed using a Transwell assay. The migration and invasion of SiHa transfected with siWTX-2# was increased compared with that of cells transfected with siRNA-control. All values are presented as the means ± SEM, **P < 0.01, ***P < 0.001, ****P < 0.0001.

To characterize the association between miR-4524b-5p and WTX. We transfected SiHa cells with three siRNAs designed to specifically silence WTX expression. The silencing efficiency was detected by qRT-PCR and western blotting, using a scrambled RNA sequence as a negative control (siRNA-control). siWTX-2# (*p* = 0.0012) could effectively decrease the expression of WTX, and we used this siRNA in further experiments (Fig 5A and 5B). In addition, we found that the expression of β-catenin mRNA and protein levels increased after WTX decreased (Fig 5C and 5D). Transwell assays and wound-healing assays showed that the cellular migration and invasion ability of SiHa cells transfected with siWTX-2# was significantly increased (Fig 5E and 5F).

### Overexpressed WTX suppressed the migration and invasion of cervical cancer cells

**Fig 6:**
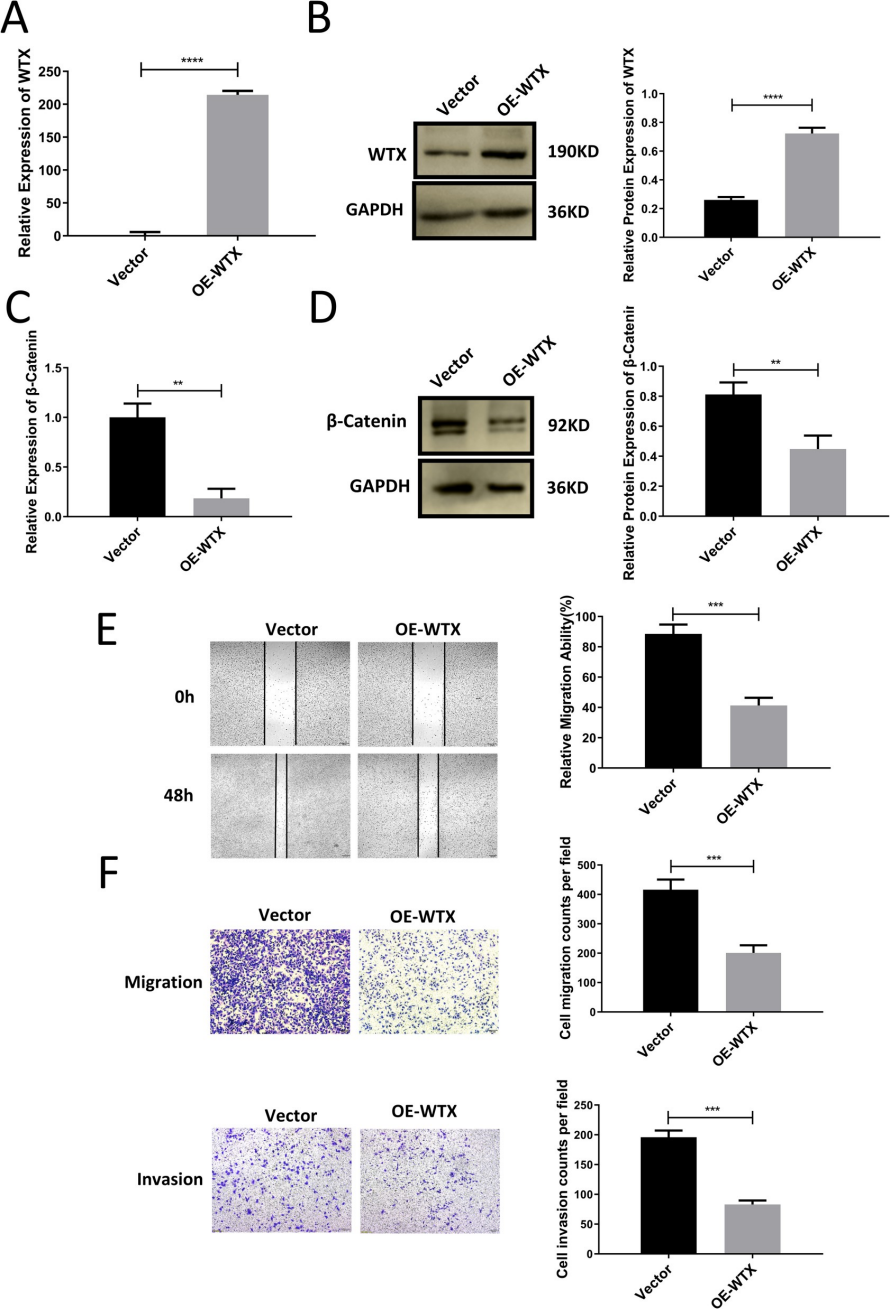
Overexpressed WTX suppressed cervical cancer cell migration and invasion. (A) Changes in WTX mRNA abundance were determined by qRT-PCR at 48 h post WTX overexpression plasmid transfection in SiHa cells. (B) Western blot analysis of WTX at 48 h post WTX overexpression plasmid transfection in SiHa cells, and the relative expression of WTX proteins were normalized to GAPDH. (C) Changes in β-catenin mRNA abundance was determined by qRT-PCR at 48 h post WTX overexpression plasmid transfection in SiHa cells. (D) Western blot analysis of β-catenin at 48 h post WTX overexpression plasmid transfection in SiHa cells, and the relative expression of β-catenin proteins were normalized to GAPDH. (E) Cell migration was analyzed using a wound-healing assay. The migration of SiHa transfected with WTX overexpression plasmid was decreased compared with that of cells transfected with control. (F) Cell migration and invasion were analyzed using a Transwell assay. The migration of SiHa transfected with WTX overexpression plasmid was decreased compared with that of cells transfected with control. All values are presented as the means ± SEM, **P < 0.01, ***P < 0.001, ****P < 0.0001.

We constructed WTX overexpression plasmid and transiently transfected into SiHa cells. We determined the overexpression efficiency by qRT-PCR and western blotting in SiHa cells. Compared to the control, the WTX overexpression plasmid dramatically increased WTX expression (Fig 6A and 6B), and decreased β-catenin expression (Fig 6C and 6D). Transwell assays and wound-healing assays showed that the cellular migration and invasion ability of SiHa cells transfected with WTX overexpression plasmid was significantly decreased (Fig 6E and 6F).

### Rescue assays showed miR-4524b-5p promoted the cell migration and invasion of cervical cancer cells by targeting the WTX gene

**Fig 7:**
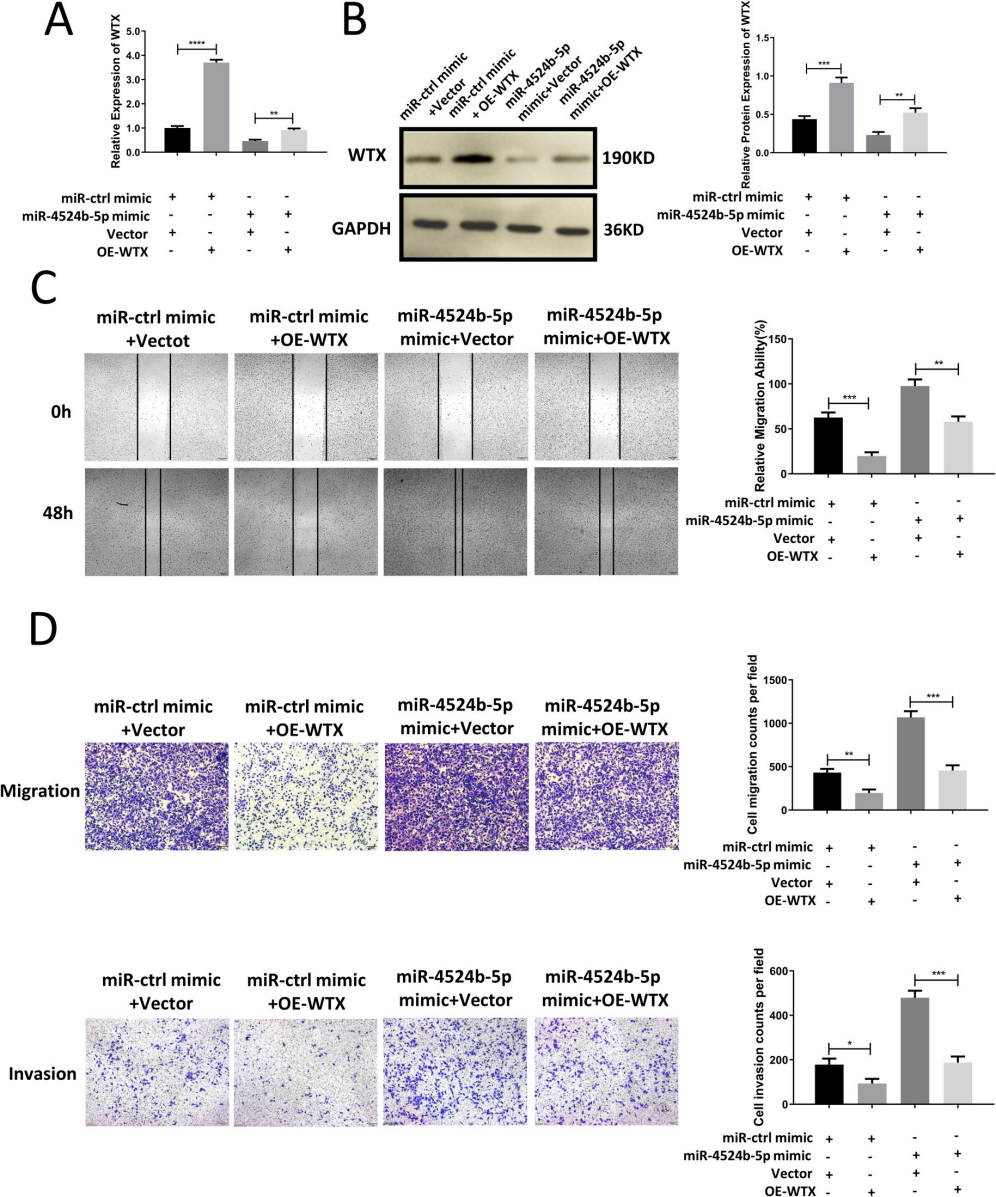
MiR-4524b-5p promoted cervical cancer cell migration and invasion by inhibiting WTX. (A) Cotransfection of the miR-4524b-5p mimic and WTX overexpression plasmid into SiHa. qRT-PCR analysis of the mRNA expression levels of WTX. (B) Cotransfection of the miR-4524b-5p mimic and WTX overexpression plasmid into SiHa. Western blot analysis of the protein expression levels of WTX. (C) Cell migration was analyzed using a wound-healing assay. (D) Cell migration and invasion were analyzed by Transwell assay. All data are presented as the means ± SEM, **P < 0.01, ***P < 0.001, ****P < 0.0001.

To further prove whether WTX was the intermediary molecule mediating miR-4524b-5p promotion of cervical cancer cell migration and invasion. We transfected miR-4524b-5p mimic and WTX overexpression plasmid into SiHa cells (Fig 7A and 7B). Transwell assays and wound-healing assays showed that miR-4524b-5p promoted the migration and invasion ability of SiHa cells, and overexpression of WTX reversed the effect of the mimic (Fig 7C and 7D). Taken together, these data suggest that WTX is involved in miR-4524b-5p-induced cervical cancer metastasis and that WTX is a functional target of miR-4524b-5p.

### WTX expression is correlated with better overall survival, and an inverse correlation is observed between miR-4524b-5p and WTX expression in cervical cancer patients

**Fig 8:**
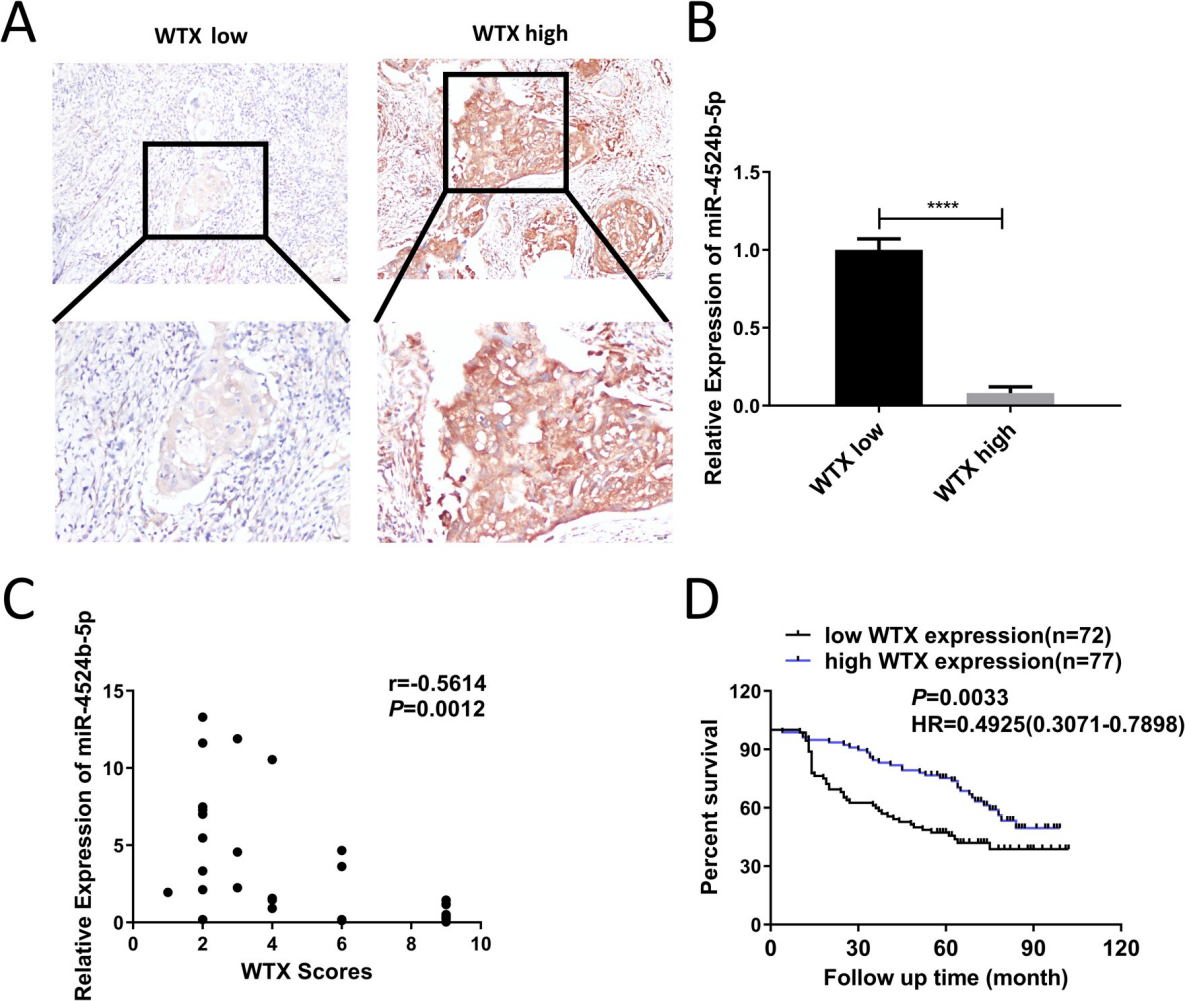
Elevated expression of WTX is linked to the improved overall survival of cervical cancer patients. (A) Representative images of IHC staining for WTX expression in cervical cancer patients. (B) qRT-PCR detected the miR-4524b-5p expression in the same cervical cancer patients. (C) The staining score of WTX was inversely correlated with miR-4524b-5p levels in the 30 cervical cancer tissue samples. (Spearman’s correlation analysis, r = -0.5614, p = 0.0012). (D) Kaplan-Meier overall survival curves for cervical patients with high or low WTX expression. All data are presented as the means ± SEM, ****P < 0.0001.

To study the relationship between WTX and cervical cancer patients, we detected the expression of WTX by IHC in 149 cervical cancer patients diagnosed at Hunan Cancer Hospital from 2005 to 2012. The low expression of WTX showed weak staining with high expression of miR-4524b-5p, while the high expression of WTX showed dark brown staining with low expression of miR-4524b-5p (Fig 8A and 8B). IHC scores revealed that WTX was negatively associated with miR-4524b-5p expression (Fig 8C, r = -0.5614, *p* = 0.0012). Thus, we demonstrated that WTX expression was inversely correlated with the expression of miR-4524b-5p in human cervical cancer. According to the staining of WTX, the 149 cases were divided into two groups. Kaplan-Meier survival analysis showed that patients with higher WTX expression were associated with a longer overall survival rate than those with lower WTX expression (Hazard Ratio = 0.4925 (0.3071–0.7898), Log-Rank *P* = 0.0033, median survival month = 84 vs. 50.5; Fig 8D).

## Discussion

Previous studies have shown that miRNAs can function as tumor suppressors or promoters in human cancers. For example, miR-148a-3p suppresses the invasive and proliferative capacity of epithelial ovarian cancer by targeting c-Met[26]. MiR-337-3p functioned as a tumor suppressor in hepatocellular carcinoma cells by targeting JAK2[27]. MiR-544 promotes cell proliferation and invasion in colorectal cancer progression by targeting forkhead box O1[28]. MiR-92a promotes cell viability and invasion via directly targeting Dickkopf-related protein 3 in cervical cancer [29]. Until recently, miR-4524b-5p has not been studied in any tumors. In this study, we detected the role of miR-4524b-5p in cervical cancer. Microarray analysis detected miR-4524b-5p upregulation in relapsed cervical cancer specimens and we verified this result in more primary and relapsed tissues by qPCR. Consistent with tissue results, the higher expression of miR-4524b-5p was detected in cervical cancer cell lines, HeLa, SiHa and me-180. Subsequently, we studied some functions of miR-4524b-5p. We found that miR-4524b-5p promoted the migration and invasion of cancer cells in vitro and the metastasis of cancer cells in vivo, but there was no effect on proliferation ability of cervical cancer cells. Thus, we provide important evidence that miR-4524b-5p function as an onco-miR in cervical cancer.

To explore the potential mechanism of miR-4524b-5p in cervical cancer. We used two online predicting algorithms TargetScan and miRanda, which showed that the WTX gene is a direct target substrate of miR-4524b-5p in cervical cancer cells, and demonstrated that suppressing or promoting the expression of miR-4524b-5p on cervical cancer cells could regulate the expression of WTX. WTX is the first gene located on the X chromosome that directly acts as a tumor suppressor[30]. WTX expression has been demonstrated to be suppressed in kidney cancer[31]. However, no one has studied the role of WTX in cervical cancer. Our research showed that the upregulation of miR-4524b-5p expression leads to the downregulation of WTX expression in cervical cancer cells. The dual luciferase reporter assay strongly demonstrated that the WTX gene was a direct target of miR-4524b-5p. The abnormal expression of miR-4524b-5p inhibited WTX expression, and WTX could attenuate miR-4524b-5p-mediated migration and invasion promotion effects on cervical cancer cells.

In the nucleus, WTX, β-catenin, β-TrCP2, APC and AXIN1 form a new complex, and this complex could inhibit the cancer process by promoting the degradation and ubiquitination of β-catenin protein and down regulating Wnt signaling[23, 30, 32, 33]. As the first tumor suppressor gene located on the X chromosome, WTX gene mutation or inactivation may trigger male X or female activation X chromosome abnormalities and prompt tumor formation[23]. We found that WTX could regulate the expression of β-catenin in cervical cancer. Moreover, Kaplan-Meier analysis verified that the higher the expression level of WTX was, the longer median overall survival time the cervical patients had. These results indicated that the expression of WTX is associated with the malignant degree of cervical cancer.

These findings suggest that the miR-4524b-5p-WTX-β-catenin axis might be a potential therapeutic target in the treatment of cervical cancer.

## Conclusions

In the present study, we identified the role of miR-4524b-5p and its mechanism of promoting cervical cancer metastasis through its direct target WTX, and miR-4524b-5p might become a key and potential biomarker in patients with cervical cancer.

## Supporting information

S1 Table MiR-4524b-5p expression levels in cervical cancer cell and normal cell lines. Relative expression of miR-4524b-5p in cervical cancer cell lines and normal cell lines. All values are presented as the means ± SEM.
(XLSX)

S2 Table MiR-4524b-5p expression levels in SiHa cells. Relative expression of miR-4524b-5p in SiHa transfected with miR-4524b-5p mimic or inhibitor. All values are presented as the means ± SEM.
(XLSX)

S3 Table WTX expression levels in cervical cancer cell and normal cell lines. Relative expression of WTX in cervical cancer cell lines and normal cell lines. All values are presented as the means ± SEM.
(XLSX)

S4 Table β-Catenin expression levels in SiHa cells. Relative expression of β-catenin in SiHa cells transfected with WTX overexpression plasmid or vector. All values are presented as the means ± SEM.
(XLSX)

S1 Fig WTX expression levels in SiHa cells. WTX expression in SiHa cells transfected with siWTX-1#, 2#, 3# and siRNA-control. Changes in protein abundance were determined by western blotting.
(TIF)

S2 Fig Elevated expression of WTX is linked to the improved overall survival of cervical cancer patients. Representative images of IHC staining for WTX expression in cervical cancer patients.
(TIF)
