# Correction: MiR-4524b-5p/WTX/β-catenin axis functions as a regulator of metastasis in cervical cancer

**DOI:** 10.1371/journal.pone.0226864

**Published:** 2019-12-17

**Authors:** Tong Li, Wenjuan Zhou, Yimin Li, Yaqi Gan, Yulong Peng, Qing Xiao, Chunli Ouyang, Anqi Wu, Sai Zhang, Jiaqi Liu, Lili Fan, Duo Han, Yu Wei, Guang Shu, Gang Yin

Fig 7 is incorrect. The authors have provided a corrected version here.

**Fig 7:**
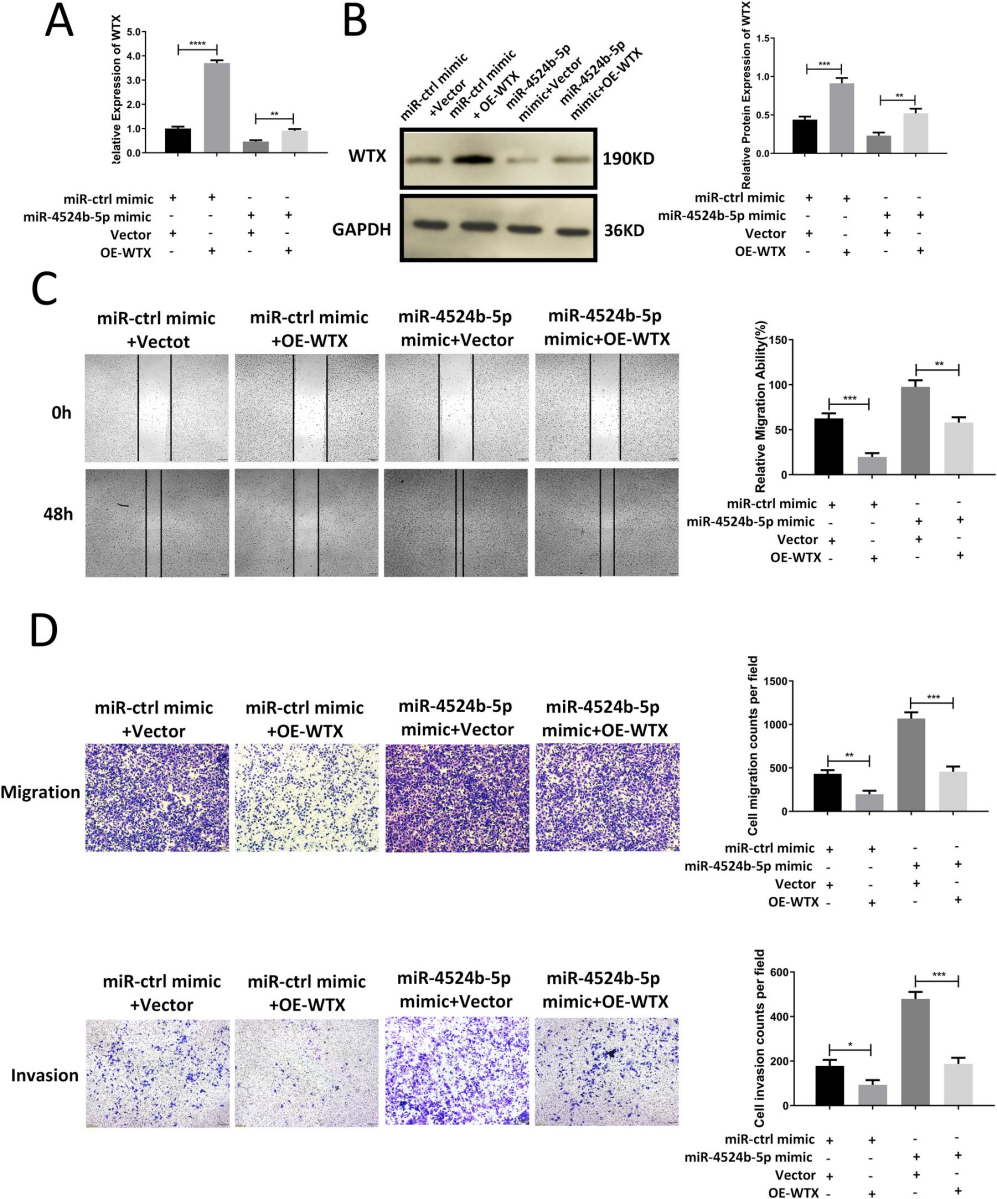
MiR-4524b-5p promoted cervical cancer cell migration and invasion by inhibiting WTX. (A) Cotransfection of the miR-4524b-5p mimic and WTX overexpression plasmid into SiHa. qRT-PCR analysis of the mRNA expression levels of WTX. (B) Cotransfection of the miR-4524b-5p mimic and WTX overexpression plasmid into SiHa. Western blot analysis of the protein expression levels of WTX. (C) Cell migration was analyzed using a wound-healing assay. (D) Cell migration and invasion were analyzed by Transwell assay. All data are presented as the means ± SEM, **P < 0.01, ***P < 0.001, ****P < 0.0001.
