# Supplementary figures and images for: MiR-4524b-5p/WTX/β-catenin axis functions as a regulator of metastasis in cervical cancer
Source: PLoS One. 2019 Apr 2;14(4):e0214822. doi: 10.1371/journal.pone.0214822 (PMC6445517; doi:10.1371/journal.pone.0214822)

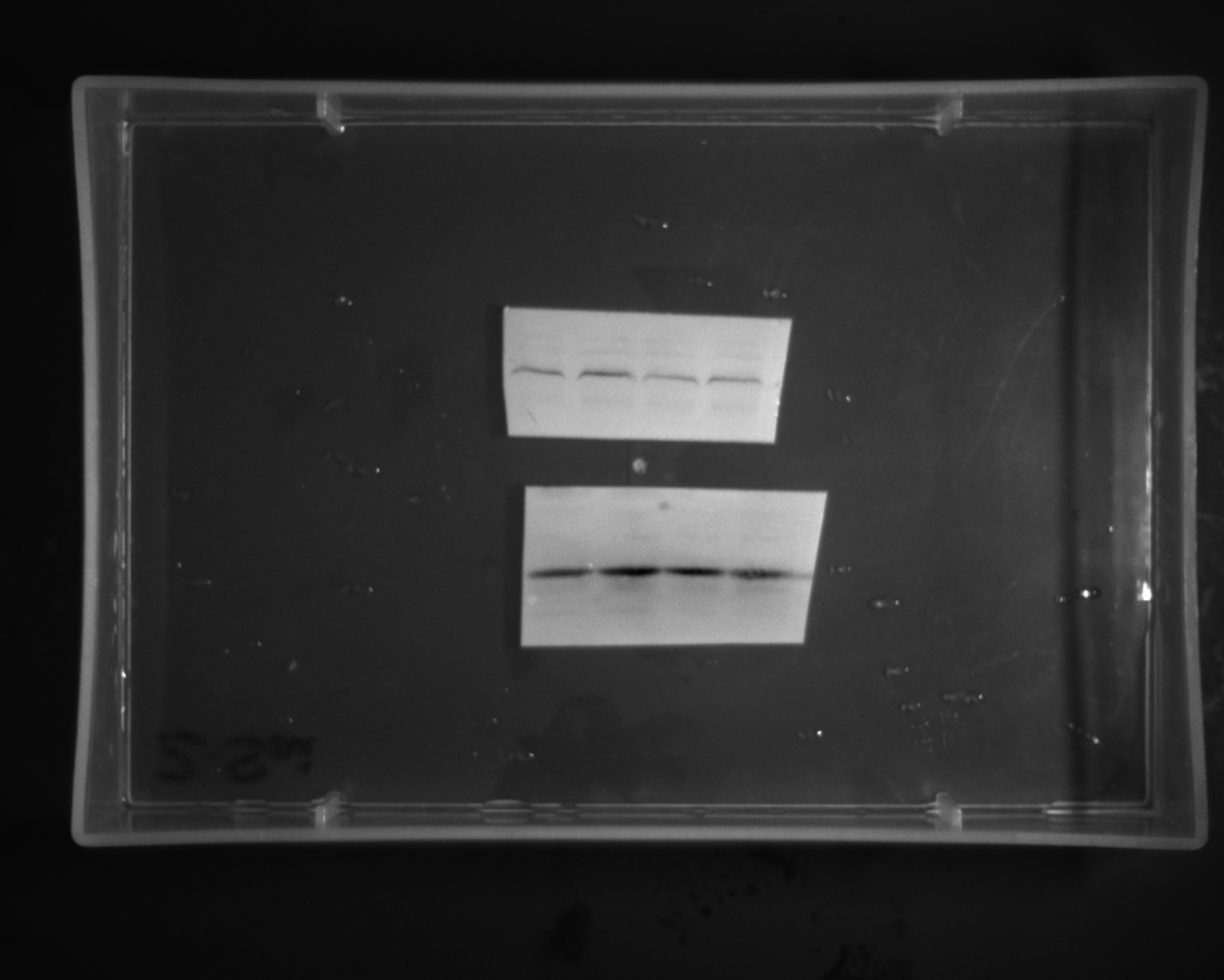

Supplement: S1 Fig — WTX expression in SiHa cells transfected with siWTX-1#, 2#, 3# and siRNA-control. Changes in protein abundance were determined by western blotting. (TIF) [file pone.0214822.s005.tif]

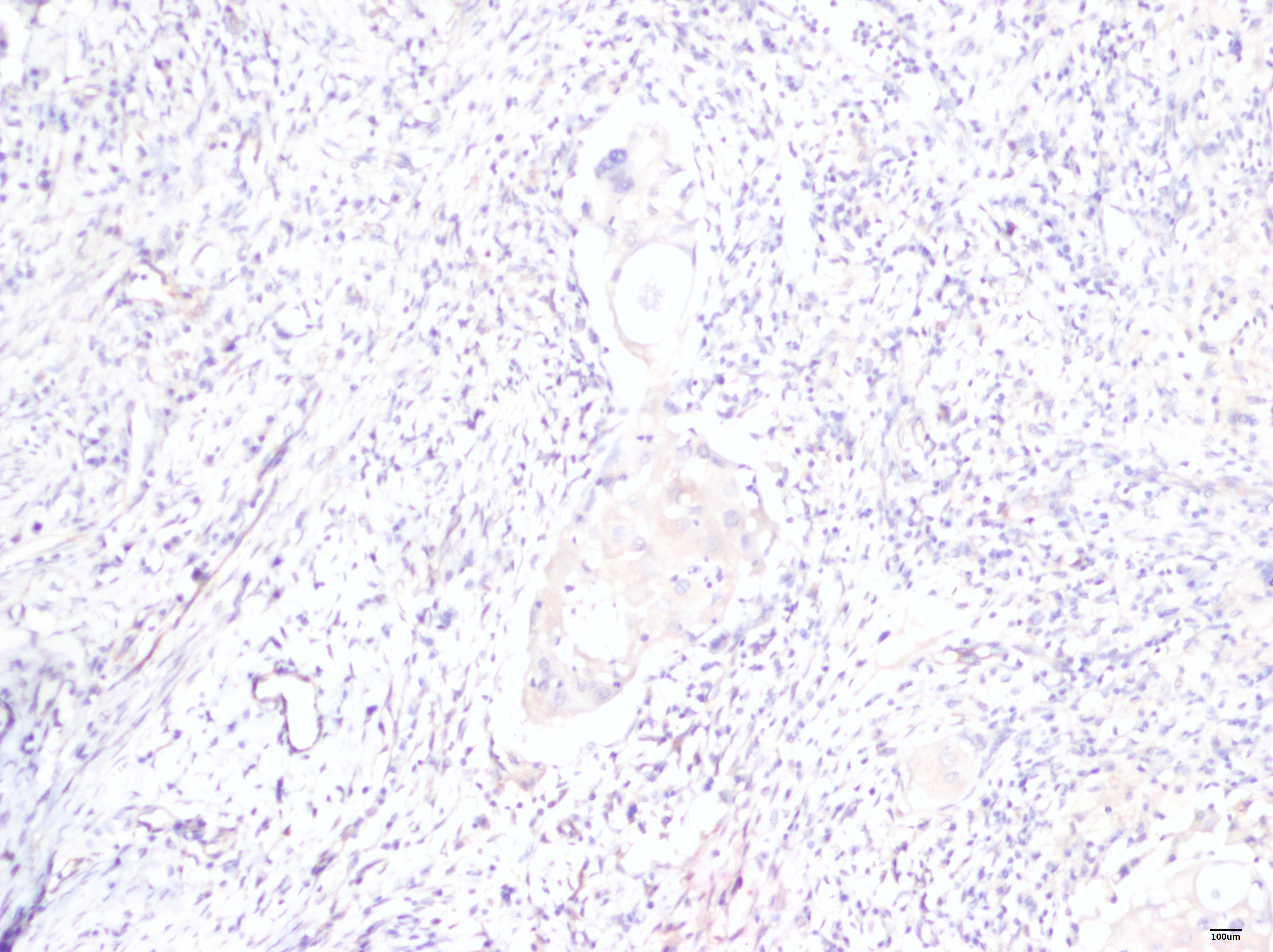

Supplement: S2 Fig — Representative images of IHC staining for WTX expression in cervical cancer patients. (TIF) [file pone.0214822.s006.tif]
